# Association of shift work with incident dementia: a community-based cohort study

**DOI:** 10.1186/s12916-022-02667-9

**Published:** 2022-12-15

**Authors:** Huanquan Liao, Dong Pan, Zhenhong Deng, Jingru Jiang, Jinhua Cai, Ying Liu, Baixuan He, Ming Lei, Honghong Li, Yi Li, Yongteng Xu, Yamei Tang

**Affiliations:** 1 Department of Neurology, Sun Yat-Sen Memorial Hospital, Sun Yat-Sen University, Guangzhou, 510120 People’s Republic of China; 2 Department of Neurology, the Seventh Affiliated Hospital, Sun Yat-Sen University, Shenzhen, 517108 People’s Republic of China; 3 Department of Neurology, the Eighth Affiliated Hospital, Sun Yat-Sen University, Shenzhen, 518033 People’s Republic of China; 4 Guangdong Provincial Key Laboratory of Malignant Tumor Epigenetics and Gene Regulation, Sun Yat-Sen Memorial Hospital, Sun Yat-Sen University, Guangzhou, 510120 People’s Republic of China; 5 Guangdong Province Key Laboratory of Brain Function and Disease, Zhongshan School of Medicine, Sun Yat-Sen University, Guangzhou, 510062 People’s Republic of China

**Keywords:** Shift work, Dementia, Alzheimer’s disease, Vascular dementia, UK Biobank

## Abstract

**Background:**

Some observational studies had found that shift work would increase risks of metabolic disorders, cancers, and cardiovascular diseases, but there was no homogeneous evidence of such an association between shift work and incident dementia. This study aimed to investigate whether shift work would increase the risk of dementia in a general population.

**Methods:**

One hundred seventy thousand seven hundred twenty-two employed participants without cognitive impairment or dementia at baseline recruited between 2006 and 2010 were selected from the UK Biobank cohort study. Follow-up occurred through June 2021. Shift work status at baseline was self-reported by participants and they were categorized as non-shift workers or shift workers. Among shift workers, participants were further categorized as night shift workers or shift but non-night shift workers. The primary outcome was all-cause dementia in a time-to-event analysis, and the secondary outcomes were subtypes of dementia, including Alzheimer’s disease, vascular dementia, and other types of dementia.

**Results:**

In total, 716 dementia cases were observed among 170,722 participants over a median follow-up period of 12.4 years. Shift workers had an increased risk of all-cause dementia as compared with non-shift workers after multivariable adjustment (hazard ratio [HR], 1.30, 95% confidence interval [CI], 1.08–1.58); however, among shift workers, night shift work was not associated with the risk of dementia (HR, 1.04, 95% CI, 0.73–1.47). We found no significant interaction between shift work and genetic predisposition to dementia on the primary outcome (*P* for interaction = 0.77).

**Conclusions:**

Shift work at baseline was associated with an increased risk of all-cause dementia. Among shift workers, there was no significant association between night shift work and the risk of dementia. The increased incidence of dementia in shift workers did not differ between participants in different genetic risk strata for dementia.

**Supplementary Information:**

The online version contains supplementary material available at 10.1186/s12916-022-02667-9.

## Background

Dementia is a cognitive disorder that significantly interferes with independent daily activities, usually caused by neurodegenerative or cerebrovascular pathologies, and common dementia subtypes include Alzheimer’s disease (AD), dementia with Lewy bodies, vascular dementia (VD), and others [1, 2]. It was estimated that the number of all-cause dementia would reach 65 million by 2030 and 113 million by 2050 worldwide [3]. For decades, unfortunately, trials aiming to treat dementia have mostly ended with failure [4]. In the absence of effective therapeutic agents, the risk factors controlling is crucial for the primary and secondary preventions of dementia [5]. Various genetic and environmental risk factors have been found that would contribute to the development of dementia, such as apolipoprotein E ε4-carriers [6], obesity [7], diabetes [8], and unhealthy lifestyles (e.g., smoking, alcohol consumption, and lack of physical activity) [9–11].

Shift work, where an individual’s normal hours of work are, in part, outside the period of the normal day working and disrupting the circadian rhythm, has become increasingly common with socioeconomic development [12]. Shift work is usually accompanied by long-hour nature, low income, a bad working environment, and increased subjective strains [13, 14], and may result in a series of health problems. Prior studies have found that shift work was associated with a 23% increased risk of myocardial infarction [15], a ~ 20% increased risk of breast cancer [16, 17], a 9–40% increased risk of type 2 diabetes [18, 19], and a 5% increased risk of ischemic stroke [15], some of which could contribute to the development of dementia. Moreover, recent studies reported that acute sleep deprivation would lead to increased brain β-amyloid (Aβ) burden and blood levels of t-tau [20, 21], from which it could be inferred that long-term shift work might lead to sleep disturbances thereby leaving those workers with a higher incidence of dementia. Taking these negative impacts into consideration, shift work may be an important risk factor for dementia.

However, there was no homogeneous evidence about the association between shift work and incident dementia [22]. The Danish Nurse Cohort Study by Jørgensen et al., involving more than 8000 nurses from 1993 to 2018, showed that persistent night shift work may increase the risk of dementia [23]. Whereas, another cohort study by Nabe-Nielsen et al., involving 4766 male employees in Denmark from 1970 to 2014, found no significant association between shift work or long working hours and the risk of dementia [24]. Most previous studies had recruited gender- or occupation-specific participants, and evidence from a more general population would be needed to investigate the relationship between shift work and incident dementia. In addition, genetic predisposition to dementia may interact with environmental factors and alter the association between shift work and dementia, and competing events that have never been considered in previous studies (e.g., death), might lead to underestimating or overestimating the impact of shift work on dementia.

Accordingly, we conducted a community-based cohort study in UK Biobank to address whether shift work would increase the risk of all-cause dementia or dementia subtypes in a general population.

## Methods

### Data source and participants

For this community-based cohort study, data were extracted from the public UK Biobank Resource [25]. The UK Biobank is a prospective cohort study with over 500,000 community-dwelling participants across the UK aged 37–73 years when recruited between 2006 and 2010 [26].

Participants who indicated they were in paid employment or self-employed at baseline were included in our study. We excluded those who (1) reported previous cognitive impairment or dementia, (2) lack of information about shift work or night shift work status, and (3) have no genetic data.

### Shift work definition

The definition of shift work in UK Biobank was “a schedule falling outside of 9 am to 5 pm; by definition, such schedules involved afternoon, evening, or night shifts or rotating through these shifts,” while night shift work was defined as “a work schedule that involves working through the normal sleeping hours, for instance, working through the hours from 12 to 6 am.”

The UK Biobank first asked participants employed at baseline to report whether their current main job involved shift schedule; if so, participants were further asked if night shifts were involved. For both questions, response options were never/rarely, sometimes, usually, or always. We derived individual current shift work status according to responses to the two questions, and categorized them as “non-shift workers” or “shift workers,” with “non-shift workers” defined as working between hours 9 am to 5 pm; among shift workers, participants were categorized as “night shift workers” or “shift but non-night shift workers”, with “non-night shift workers” defined working between hours 5 pm to 12 am; among night shift workers, participants were further categorized as “some night shift workers” or “usual/permanent night shift workers.”

### Outcomes

The primary outcome was all-cause dementia in a time-to-event analysis, and the secondary outcomes included AD, VD, and other types of dementia. The electronic health records (EHRs), a data linkage to hospital inpatient admissions and death registries, include primary or secondary events in England, Scotland, and Wales. A previous comparison between EHRs and expert clinical adjudicators in the UK Biobank showed that the overall positive predictive value for dementia diagnosis is 82.5% [27], suggesting that the EHRs were effective to assess the association between risk factors and dementia. We used the algorithms provided by UK Biobank to identify dementia cases, which were generated based on EHRs, using ICD-9 and ICD-10 codes (Additional file 1: Table S1). In the time-to-event analysis, the date of incident dementia during follow-up was set as the earliest date of dementia codes recorded regardless of the source used. At the time of analysis, as hospital admission data were available until 30 June 2021, we, therefore, censored the disease-specific outcome analysis at this date or the date of the first disease incidence or death, whichever occurred first. Mortality data were available for participants until 31 May 2021.

### Polygenetic risk score for dementia

We developed a polygenetic risk score (PRS) for quantifying the genetic predisposition to dementia using single-nucleotide polymorphisms (SNPs) associated with dementia based on previous genome-wide association studies that did not include UK Biobank participants [28]. Information on the 23 selected SNPs is listed in Additional file 1: Table S2. Individual SNPs were coded as 0, 1, and 2 according to the number of risk alleles. The PRS was formulated as the sum of the number of risk alleles at each locus multiplied by the respective regression coefficient, divided by the number of SNPs, using PRSice-2 [29, 30]. The PRS was then divided into quartiles and categorized as low (quartiles 1 to 2), intermediate (quartile 3), and high (quartile 4) genetic predisposition to dementia (Additional file 1: Table S3).

### Covariates

Possible confounding variables include: age; sex; ethnicity (white/not white); education, categorized as higher (college/university degree or other professional qualification), upper secondary (second/final stage of secondary education), lower secondary (first stage of secondary education), vocational (work-related practical qualifications), or other; socioeconomic status, categories derived from Townsend deprivation index quartiles 1 (low), 2 to 3 (intermediate), and 4 (high); diabetes mellitus (DM); hypertension (HTN); stroke; coronary heart disease (CHD); cholesterol-lowering medication; antihypertensives; aspirin; body mass index (BMI); systolic blood pressure (SBP); total cholesterol (TC); triglycerides (TG); high-density lipoprotein (HDL); low-density lipoprotein (LDL); glycated hemoglobin (HbA1c); smoking status (current or no current smoking); alcohol consumption; healthy diet, based on consumption of at least 4 of 7 commonly eaten food groups following recommendations on dietary priorities [31]; regular physical activity, defined as meeting the 2017 UK Physical activity guidelines of 150 min of moderate activity per week or 75 min of vigorous activity; years of work; sleep duration, categorized as ≤ 6, 7–8, and ≥ 9 h/day; chronotype preference (definitely a “morning” person, more a “morning” than “evening” person, more an “evening” than a “morning” person, and definitely an “evening” person).

### Statistical analysis

For baseline characteristics, continuous variables conforming to normal distribution were described by their means and standard deviations, while those not conforming to normal distribution were described by medians and interquartile ranges. Categorical variables were described by counting numbers and calculating percentages. Univariate comparisons between groups were performed using Student’s *t*, Mann–Whitney, or *χ*^2^ tests according to the type and distribution of variables.

In the primary analysis, time-to-event analysis for all-cause dementia was performed using the Cox proportional hazard regression model, and we constructed several models that included different covariates to estimate hazard ratios (HR) and their 95% confidence intervals (95% CI). Model 1 was adjusted for age at baseline and sex. Model 2 was adjusted for terms in model 1, ethnicity, education, and socioeconomic status. Model 2 was chosen as the primary model.

We used a fixed sequence procedure for multiple comparisons, which would not inflate the type I error. We sequentially compared differences in the incidence of dementia between shift workers and non-shift workers, night shift workers and shift but non-night shift workers, and some/usual night shift workers and permanent night shift workers. In the subgroup analysis, which was set out to explore whether the impact of shift work on dementia varied in the subgroups defined according to age at baseline (≤ 60, > 60 years), ethnicity, sex, socioeconomic status, sleep duration, and genetic predisposition to dementia by PRS, the *P* value for interaction was calculated by the tests of exposure-by-covariate interaction in the Cox models. The secondary outcomes of dementia subtypes were analyzed using the same Cox models of the primary analysis.

We conducted several sensitivity analyses. First, we further adjusted some covariate. Model 3 was further adjusted for terms in model 2, DM, HTN, stroke, CHD, cholesterol-lowering medication, antihypertensives, aspirin, BMI, SBP, TC, TG, HDL, LDL, HbA1c, smoking status, alcohol consumption, healthy diet, and regular physical activity. Model 4 was adjusted for terms in model 3, genetic predisposition to dementia by PRS category. Model 5 was adjusted for terms in model 4, years of work. Model 6 was adjusted for terms in model 5, sleep duration. Model 7 was adjusted for terms in model 6, chronotype preference. Second, we analyzed the impact of shift work on dementia using Fine-Gray methods accounting for death as a competing risk, to assess the robustness of our findings [32]. Third, we also excluded subjects with follow-up time < 1 year or incident dementia < 1 year from baseline to perform the analysis. Forth, we perform the same analysis in the dataset containing 278,270 participants using multiple imputations by chained equations with 5 imputations to impute missing values.

All *P* values were reported as two-sided tests with significance defined as *P* < 0.05. Statistical analyses were performed in the R software (Version 4.0.3, R Core Team, https://www.r-project.org).

## Results

### Baseline characteristics

**Fig. 1:**
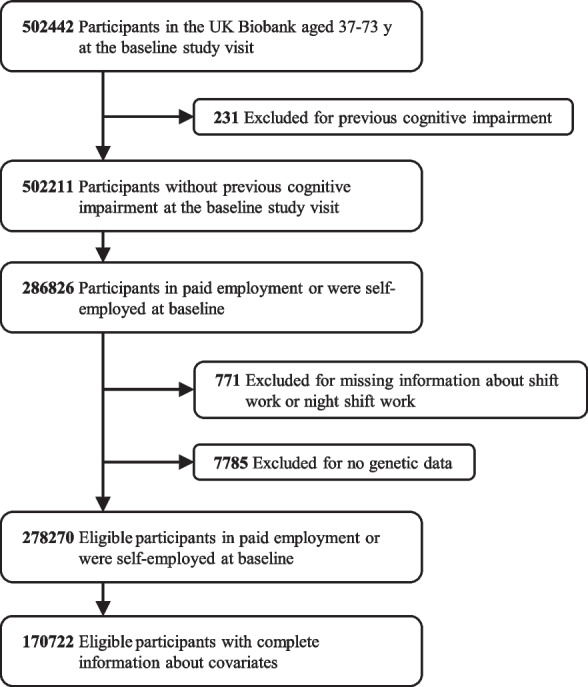
Flowchart of participants throughout the study

Figure 1 has illustrated the participants’ selection. Of 170,722 eligible participants, 27,450 (16.1%) had reported shift work status and 143,272 (83.9%) had not (non-shift workers). Baseline characteristics of eligible participants were displayed in Tables 1 and 2. Participants who had reported shift work status (vs. non-shift workers) were younger, more likely to be men, had a lower education level and higher Townsend deprivation index, and had a higher prevalence of DM and HTN. Shift workers also tended to take more cholesterol-lowering medication, antihypertensives, and aspirin, and to be not current smokers, had lower alcohol consumption, less healthy diet, but more physically active and had a shorter sleep duration. (Table 1).

**Table 1:** Baseline characteristics of the study participants according to shift work status

Characteristics	Current work schedule	
	**Non-shift workers**	**Shift workers**
Sample size, *n*	143,272	27,450
Age (year)	52.8 ± 7.1	51.8 ± 7.0
Sex/female	74,896 (52.3)	12,010 (43.8)
Ethnicity/White	136,909 (95.6)	24,963 (90.9)
Education		
Higher	81,636 (57.0)	12,200 (44.4)
Upper secondary	12,506 (8.7)	2064 (7.5)
Lower secondary	23,551 (16.4)	5039 (18.4)
Vocational	9158 (6.4)	3163 (11.5)
Other	16,421 (11.5)	4984 (18.2)
Townsend deprivation index category		
Low	38,512 (26.9)	5318 (19.4)
Intermediate	73,149 (51.1)	13,143 (47.9)
High	31,611 (22.1)	8989 (32.7)
DM	4719 (3.3)	1227 (4.5)
HTN	29,561 (20.6)	6151 (22.4)
Stroke	1150 (0.8)	233 (0.8)
CHD	3205 (2.2)	775 (2.8)
Cholesterol-lowering medication	15,025 (10.5)	3222 (11.7)
Antihypertensives	19,491 (13.6)	4140 (15.1)
Aspirin	13,648 (9.5)	2826 (10.3)
BMI (kg/m^2^)	27.05 ± 4.59	27.96 ± 4.84
SBP (mmHg)	134.69 ± 17.75	135.23 ± 17.48
TC (mmol/L)	5.69 ± 1.08	5.65 ± 1.08
TG (mmol/L)	1.68 ± 1.02	1.80 ± 1.11
HDL (mmol/L)	1.45 ± 0.37	1.38 ± 0.36
LDL (mmol/L)	3.56 ± 0.83	3.56 ± 0.83
HbA1c (%)	5.36 ± 0.54	5.43 ± 0.62
No current smoking	129,339 (90.3)	23,275 (84.8)
Alcohol consumption at least weekly	106,793 (74.5)	18,306 (66.7)
Healthy diet	91,682 (64.0)	16,019 (58.4)
Regular physical activity	72,331 (50.5)	16,702 (60.8)
Genetic predisposition to dementia by PRS category		
Low	71,309 (49.8)	13,546 (49.3)
Intermediate	36,223 (25.3)	7226 (26.3)
High	35,740 (24.9)	6678 (24.3)
Years of work (years)	12.85 ± 10.65	13.15 ± 10.66
Sleep duration category (h/day)		
≤ 6	34,289 (23.9)	8690 (31.7)
7–8	102,900 (71.8)	17,337 (63.2)
≥ 9	6083 (4.2)	1423 (5.2)
Chronotype		
Definitely a “morning” person	37,047 (25.9)	7245 (26.4)
More a “morning” than “evening” person	52,168 (36.4)	8763 (31.9)
More an “evening” than a “morning” person	41,123 (28.7)	8337 (30.4)
Definitely an “evening” person	12,934 (9.0)	3105 (11.3)

Data are means ± standard deviations, or *N* (%)

*Abbreviation*:* DM* diabetes mellitus, *HTN* hypertension, *CHD* coronary heart disease, *BMI* body mass index, *SBP* systolic blood pressure, *TC* total cholesterol, *TG* triglycerides, *HDL* high-density lipoprotein, *LDL* low-density lipoprotein, *HbA1c* glycated hemoglobin, *PRS* polygenic risk score

**Table 2:** Baseline characteristics of shift workers according to night shifts work status

Characteristics	Shift workers	
	**Shift but non-night shift workers**	**Night shift workers**
Sample size, n	13,729	13,721
Age (year)	52.39 ± 7.06	51.18 ± 6.85
Sex/female	7036 (51.2)	4974 (36.3)
Ethnicity/White	12,676 (92.3)	12,287 (89.5)
Education		
Higher	6234 (45.4)	5966 (43.5)
Upper secondary	1090 (7.9)	974 (7.1)
Lower secondary	2520 (18.4)	2519 (18.4)
Vocational	1435 (10.5)	1728 (12.6)
Other	2450 (17.8)	2534 (18.5)
Townsend deprivation index category		
Low	2676 (19.5)	2642 (19.3)
Intermediate	6643 (48.4)	6500 (47.4)
High	4410 (32.1)	4579 (33.4)
DM	582 (4.2)	645 (4.7)
HTN	3041 (22.2)	3110 (22.7)
Stroke	129 (0.9)	104 (0.8)
CHD	370 (2.7)	405 (3.0)
Cholesterol-lowering medication	1600 (11.7)	1622 (11.8)
Antihypertensives	2045 (14.9)	2095 (15.3)
Aspirin	1411 (10.3)	1415 (10.3)
BMI (kg/m^2^)	27.70 ± 4.91	28.21 ± 4.76
SBP (mmHg)	134.84 ± 17.65	135.63 ± 17.30
TC (mmol/L)	5.66 ± 1.07	5.63 ± 1.09
TG (mmol/L)	1.74 ± 1.06	1.87 ± 1.15
HDL (mmol/L)	1.42 ± 0.37	1.35 ± 0.35
LDL (mmol/L)	3.56 ± 0.83	3.56 ± 0.84
HbA1c (%)	5.42 ± 0.61	5.44 ± 0.63
No current smoking	11,871 (86.5)	11,404 (83.1)
Alcohol consumption at least weekly	9218 (67.1)	9088 (66.2)
Healthy diet	8297 (60.4)	7722 (56.3)
Regular physical activity	8109 (59.1)	8593 (62.6)
Genetic predisposition to dementia by PRS category		
Low	6804 (49.6)	6742 (49.1)
Intermediate	3596 (26.2)	3630 (26.5)
High	3329 (24.2)	3349 (24.4)
Years of work (years)	12.23 ± 10.34	14.07 ± 10.90
Sleep duration category (h/day)		
≤ 6	3956 (28.8)	4734 (34.5)
7–8	9054 (65.9)	8283 (60.4)
≥ 9	719 (5.2)	704 (5.1)
Chronotype		
Definitely a “morning” person	3835 (27.9)	3410 (24.9)
More a “morning” than “evening” person	4633 (33.7)	4130 (30.1)
More an “evening” than a “morning” person	4013 (29.2)	4324 (31.5)
Definitely an “evening” person	1248 (9.1)	1857 (13.5)

Data are means ± standard deviations, or *N* (%)

*Abbreviation*:* DM* diabetes mellitus, *HTN* hypertension, *CHD* coronary heart disease, *BMI* body mass index, *SBP* systolic blood pressure, *TC* total cholesterol, *TG* triglycerides, *HDL* high-density lipoprotein, *LDL* low-density lipoprotein, *HbA1c* glycated hemoglobin, *PRS* polygenic risk score

### Shift work or night shift work and dementia

**Fig. 2:**
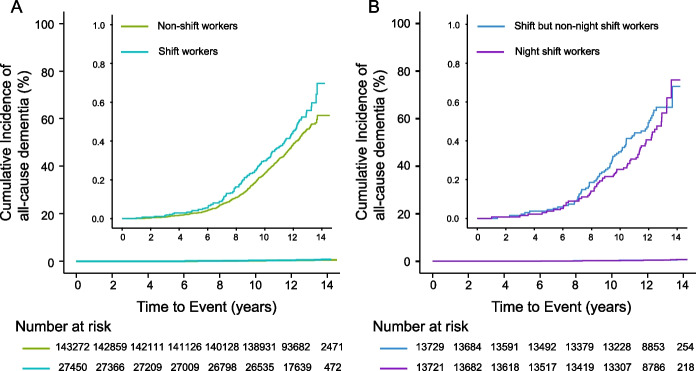
Kaplan–Meier survival analyses for all-cause dementia between non-shift workers and shift workers (**A**), shift but non-night shift workers and night shift workers (**B**)

The incidence of the primary and secondary outcomes was shown in Additional file 1: Table S4. We observed 716 dementia cases during a median follow-up period of 12.4 years, of whom 134 (18.7%) and 582 (81.3%) were in the shift workers group and the non-shift workers group, respectively. Shift workers had a higher incidence of all-cause dementia compared with non-shift workers (unadjusted-HR, 1.21; 95% CI, 1.00 to 1.46; *P* = 0.04; Fig. 2). After adjusting for confounders, the risk of all-cause dementia among shift workers remained significantly higher than non-shift workers (adjusted-HR, 1.30; 95% CI, 1.08 to 1.58; *P* = 0.006; Table 3). Among shift workers, we did not observe a significant association between night shift work and the risk of dementia after multivariable adjustment in the Cox model (adjusted-HR, 1.04; 95% CI, 0.73 to 1.47; *P* = 0.83; Table 3), and the sensitivity analysis yielded similar results (Additional file 1: Table S5-8).

**Table 3:** Hazard ratios for primary outcome associated with current work schedule

Sequency	Groups	Sample size	Cases	Person-years	Incidence rate per 100,000 person-years	Model 1	Model 2
First	Non-shift workers	143,272	582	1,753,205	33.19	Ref	Ref
	Shift workers	27,450	134	335,034	39.99	1.41 [1.17, 1.70]	1.30 [1.08, 1.58]
	*P* value	-	-	-	-	< 0.001	0.006
Second	Shift but non-night shift workers	13,729	71	167,423.4	42.40	Ref	Ref
	Night shift workers	13,721	63	167,610.6	37.58	1.09 [0.77, 1.53]	1.04 [0.73, 1.47]
	*P* value	-	-	-	-	0.63	0.83
Third	Some night shift workers	7915	32	96,623.63	33.11	Ref	Ref
	Usual/permanent night shift workers	5806	31	70,986.94	43.67	1.32 [0.81, 2.17]	1.25 [0.76, 2.07]
	*P* value	-	-	-	-	0.26	0.37

Data are hazard ratios (95% confidence interval). Model 1 was adjusted for age at baseline and sex. Model 2 was adjusted for terms in model 1, ethnicity, education, and socioeconomic status

For the secondary outcomes of dementia subtypes, we found no significance of the association between shift work and the risks of AD (adjusted-HR, 1.23; 95% CI, 0.90 to 1.69; *P* = 0.20) and VD (adjusted-HR, 1.46; 95% CI, 0.94 to 2.27; *P* = 0.09) (Table 4).

**Table 4:** Hazard ratios for secondary outcome associated with current work schedule

Secondary outcomes	Groups	Sample size	Cases	Person-years	Incidence rate per 100,000 person-years	Model 1	Model 2
AD	Non-shift workers	143,272	223	1,754,038	12.71	Ref	Ref
	Shift workers	27,450	48	335,241	14.31	1.37 [1.00, 1.87]	1.23 [0.90, 1.69]
	*P* value	-	-	-	-	0.05	0.20
VD	Non-shift workers	143,272	101	1,754,274	5.75	Ref	Ref
	Shift workers	27,450	26	335,335	7.75	1.63 [1.06, 2.50]	1.46 [0.94, 2.27]
	*P* value	-	-	-	-	0.02	0.09
Other types of dementia	Non-shift workers	143,272	418	1,753,631	23.83	Ref	Ref
	Shift workers	27,450	99	335,172	29.53	1.44 [1.15, 1.79]	1.34 [1.07, 1.68]
	*P* value	-	-	-	-	0.001	0.01

*Abbreviations: AD* Alzheimer’s disease, *VD* vascular dementia

Data are hazard ratios (95% confidence interval). Model 1 was adjusted for age at baseline and sex. Model 2 was adjusted for terms in model 1, ethnicity, education, and socioeconomic status

### Subgroup and sensitivity analyses

**Fig. 3:**
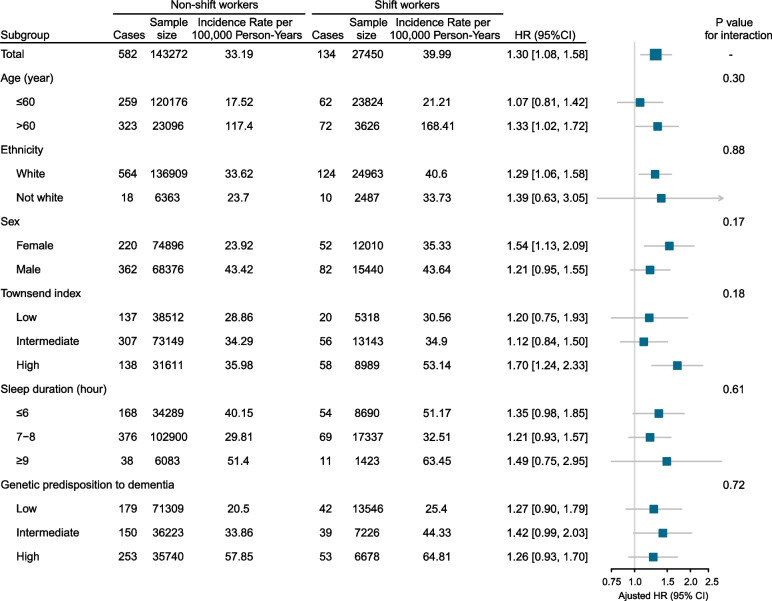
Association of shift work and the risk of all-cause dementia stratified by potential risk factors. Abbreviations: HR, hazard ratio; CI, confidence interval. Results were adjusted for age at baseline, sex, ethnicity, education, and socioeconomic status. Horizontal lines indicate the ranges of 95% CIs and the vertical dash lines indicate the hazard ratio of 1.0

As shown in Fig. 3, the impact of shift work on dementia did not differ among participants who were in the low-, intermediate-, or high-PRS subgroups (*P* for interaction = 0.77). Similarly, no significant interaction was observed in the subgroups of age at baseline, ethnicity, sex, socioeconomic status, and sleep duration.

In order to assess the robustness of our findings, we conducted several sensitivity analyses, including the models further adjusted for genetic predisposition to dementia by PRS, years of work, sleep duration, and chronotype category, the Fine-Gray methods under consideration of the competing risk of death, the models excluding subjects with follow-up time < 1 year or incident dementia < 1 year from baseline and the models of the imputed dataset. The results showed no substantial change of the impact of shift work on dementia (Additional file 1: Table S5-8).

## Discussion

In this community-based cohort study in UK Biobank, involving 170,722 participants without cognitive impairment or dementia at baseline, we found that shift workers at baseline had a 30% increased risk of all-cause dementia as compared with non-shift workers during a median follow-up period of 12.4 years; however, among shift workers, there was no significant association between night shift work and the risk of dementia. In addition, to the best of our knowledge, it was the first study to examine the interaction between shift work and genetic predisposition to dementia, and we found that the risk of dementia associated with shift work did not significantly differ among participants in different genetic risk strata of dementia.

Although some health problems that are caused by shift work may contribute to the development of dementia, such as metabolic disorders and ischemic stroke, the mechanism that how shift work causes cognitive impairment still remains unclear. We inferred that sleep disturbance and disrupted circadian might be the main causes of cognitive impairment among shift workers [33]. Systematic reviews had shown an increased risk for shift workers to develop chronic sleep disturbance [34, 35], and the prevalence of shift work sleep disorder has been estimated to be 10–23% in shift workers [36]. Extracellular levels of metabolites, including amyloid β, increase in the brain during wakefulness and are reduced during sleep, and sleep disturbances could therefore result in a reduced clearance of these metabolites [37], which contributes to the pathogenesis of AD. And cognitive performance deteriorates with sleep disturbance [38, 39].

Most regulatory hormones show strong diurnal rhythms, e.g., cortisol and melatonin, and disturbed sleep is often related to a mild temporary increase in the major neuroendocrine stress systems [40]. Experimental studies showed that disturbed sleep, altered light exposure typical for shift workers, could lead to an acute circadian disruption and so influence the normal secretion of these regulatory hormones [41]. Studies of patients with AD have found that these patients had a higher prevalence of melatonin secretion rhythm disorders [42, 43]. Animal experiments showed that melatonin can inhibit expressions of amyloid-β protein in the hippocampal area of model rats with senile dementia [44]. Activation of the type 1 melatonin receptor modulated anti-amyloidogenic and anti-inflammatory roles in AD mice brain and improved the cognitive deficits [45]. Shift work was also associated with abnormalities in brain structure that had been observed in dementia pathophysiology, giving a hint of the underlying brain mechanisms of shift work on dementia risk [46]. In addition, shift work has been linked to lower socioeconomic status, which is consistent with our results that participants who had reported shift work status (vs. non-shift workers) had higher Townsend deprivation indexes (i.e., lower socioeconomic status), and may lead to disruption of social rhythms, that is a conflict between work and family demands. Thus, shift workers may suffer from higher psychosocial work stress [33].

A systemic review by Leso et al. in 2021 found several literatures investigating the association between shift work and dementia, but failed to draw definitive conclusions on this topic, because of the limited number of available studies, a different definition of work schedules, and the possible co-exposure to other occupational risk factors [22]. The Danish Nurse Cohort Study by Jørgensen et al., involving more than 8000 nurses from 1993 to 2018, showed that persistent night shift work may increase the risk of dementia [23]. Whereas, another cohort study by Nabe-Nielsen et al., involving 4766 male employees in Denmark from 1970 to 2014, found no significant association between shift work or long working hours and the risk of dementia [24]. Previous studies have shown mixed results [47–49].

Since most previous studies had only recruited gender- or occupation-specific participants, our study, from a more general population in the UK biobank, provided strong evidence that shift work at baseline is associated with an increased risk of dementia, and extensive sensitivity analyses assessing the robustness of our findings have all yielded similar results. It should be emphasized that participants from the UK Biobank were not nationally representative due to the low response rate (~ 5.5%) and the fact that the participants who were in employment at baseline and included in our analysis tended to be healthier than those who had retired earlier, which might lead to potential healthy volunteer selection bias. However, given that the UK Biobank has a tremendous sample size and a median follow-up time of over 10 years, it still has the capacity to detect and identify risk factors [50], and our findings also remain of important public health implications in terms of the need for effective public measures to reduce the risk of dementia in order to improve the quality of life and health of shift workers, such as increasing the minimum hourly wage and reducing the frequency or duration of shift work.

Another important finding was that different genetic predispositions to dementia did not significantly alter the association between shift work and dementia, from which it could be inferred that shift workers may benefit from reducing the duration or frequency of shift work regardless of the genetic predisposition to dementia if the associations were causal. Furthermore, the subgroup analysis indicated that the impact of shift work on dementia was more pronounced in those aged 60 years and older. Considering that the age of onset of dementia is usually above 80 years [51] while the mean age at baseline of participants in our study was only 52 years and the median follow-up was 12.4 years, the association between shift work and dementia could have been underestimated. Further studies, enrolling more elderly volunteers, would be needed in the future to verify our findings.

Contrary to our expectations, among shift workers, this study did not find a statistical difference in the risk of dementia between night shift workers and non-night shift workers. This result appeared to be contrary to the idea that night shift work may lead to more severe circadian disturbances and sleep impairment, resulting in an increased risk of dementia. In fact, in our study, the proportion of participants with a sleep duration less than 6 h was higher in night shift workers compared with non-night shift workers and our results (Additional file 1: Table S9) showed that sleep duration less than 6 h was associated with an increased risk of dementia, in line with previous studies [52]. Possibly due to the small number of events in subgroups, we did not have sufficient statistical power to detect a difference. Hence, further studies with larger sample sizes of shift workers would be warranted to address whether there would be a difference in the risk of dementia between night shift workers and non-night shift workers.

Overall, our study provides novel evidence based on a general population that shift work at baseline may lead to an increased risk of dementia regardless of genetic predisposition to dementia and suggests that the occupational management of reducing the duration or frequency of shift work may be crucial for long-term shift workers.

### Strengths and limitations

Our study has several major strengths. Firstly, the large sample size and the wealth of information on lifestyle, and other covariates of UK Biobank participants, enabled this study of comprehensive sensitivity analyses and subgroup analyses. Secondly, to our best knowledge, it was the first study to examine the interaction between shift work and genetic predisposition to dementia. There were also several limitations in our study. Firstly, the study was a retrospective analysis of data from the UK Biobank, thus confounders that were included in the multivariable Cox model were based on available variables in the database and there might be some unknown or unmeasured biases confounding the association between shift work and dementia. Besides, some covariates had too much missing data, resulting in a loss of sample size. Secondly, although we believe that the UK Biobank has sufficient capacity to identify risk factors, its low response rate and healthy volunteer bias may still contribute to an underestimation of the impact of shift work on dementia, which needs to be further assessed in future studies. Thirdly, dementia might be misdiagnosed or underdiagnosed, and participants with cognitive impairment usually are more likely to be lost to follow-up, hence some dementia cases might not be captured by EHRs. Furthermore, the work schedule information was assessed only at the baseline. Participants’ work status might change over time during the follow-up while people tend to stop doing shift or night shift work at an older age, which might bias our results toward the null hypothesis, resulting in an underestimation of the effect size [53]. Future prospective studies measuring the longitudinal change of employment status would be needed to assess the association of lifetime exposure to shift work with the risk of dementia. Finally, participants recruited by UK Biobank were mostly white British, which may limit the extrapolation of our findings to other ethnicities, such as Asians and Africans.

## Conclusions

Shift workers at baseline was associated with a higher incidence of all-cause dementia compared with non-shift workers. Among shift workers, there was no significant association between night shift work and the risk of dementia. The increased incidence of dementia in shift workers did not differ between participants in different genetic risk strata for dementia. Our findings have public health implications for the primary prevention of dementia, but future prospective studies are still warranted to determine whether reducing the frequency or duration of shift work would contribute to lowering the risk of incident dementia and to clarify the underlying mechanisms.

## Supplementary Information

**Additional file1: Table S1.** Codes used in the UK Biobank study to identify dementia cases. **Table S2.** SNPs information for constructing polygenetic risk score. **Table S3.** Hazard ratios for primary outcome associated with genetic predisposition to dementia by PRS category. **Table S4.** Incidence of primary outcome and secondary outcomes. **Table S5.** Hazard ratios for primary outcome associated with current work schedule of models further adjusted. **Table S6.** Hazard ratios for primary outcome associated with current work schedule using Fine-Gray methods accounting for death as a competing risk in models. **Table S7.** Hazard ratios for primary outcome associated with current work schedule after excluding participants with follow-up time < 1 year or incident dementia <1 year from baseline. **Table S8.** Hazard ratios for primary outcome associated with current work schedule in the imputed dataset. **Table S9.** Hazard ratios for primary outcome associated with sleep duration.

## Data Availability

The UK Biobank resources are available upon reasonable request and can be accessed through applications on their website (https://www.ukbiobank.ac.uk/enable-your-research/apply-for-access), and by contacting access@ukbiobank.ac.uk. Ethics approval and consent to participate The UK Biobank Study’s ethical approval was granted by the National Information Governance Board for Health and Social Care and the NHS North West Multicentre Research Ethics Committee. All participants provided informed consent through electronic signature at baseline assessment. The present study was conducted under application number 70109 of the UK Biobank resource.

## Abbreviations

AD: Alzheimer’s disease
BMI: Body mass index
CHD: Coronary heart disease
CI: Confidence intervals
DM: Diabetes mellitus
EHRs: Electronic health records
HbA1c: Glycated hemoglobin
HDL: High-density lipoprotein
HR: Hazard ratios
HTN: Hypertension
LDL: Low-density lipoprotein
PRS: Polygenetic risk score
SBP: Systolic blood pressure
SNPs: Single-nucleotide polymorphisms
TC: Total cholesterol
TG: Triglycerides
VD: Vascular dementia
