# Supplementary material for: Association of shift work with incident dementia: a community-based cohort study
Source: BMC Med. 2022 Dec 15;20:484. doi: 10.1186/s12916-022-02667-9 (PMC9753386; doi:10.1186/s12916-022-02667-9)
Supplement: Supplementary file 1 — Additional file1: Table S1. Codes used in the UK Biobank study to identify dementia cases. Table S2. SNPs information for constructing polygenetic risk score. Table S3. Hazard ratios for primary outcome associated with genetic predisposition to dementia by PRS category. Table S4. Incidence of primary outcome and secondary outcomes. Table S5. Hazard ratios for primary outcome associated with current work schedule of models further adjusted. Table S6. Hazard ratios for primary outcome associated with current work schedule using Fine-Gray methods accounting for death as a competing risk in models. Table S7. Hazard ratios for primary outcome associated with current work schedule after excluding participants with follow-up time < 1 year or incident dementia <1 year from baseline. Table S8. Hazard ratios for primary outcome associated with current work schedule in the imputed dataset. Table S9. Hazard ratios for primary outcome associated with sleep duration. [file 12916_2022_2667_MOESM1_ESM.docx]

**Additional file 1**

**Table S1. Codes used in the UK Biobank study to identify dementia cases**

**Table S2. SNPs information for constructing polygenetic risk score**

**Table S3. Hazard ratios for primary outcome associated with genetic predisposition to dementia by PRS category**

**Table S4. Incidence of primary outcome and secondary outcomes**

**Table S5. Hazard ratios for primary outcome associated with current work schedule of models further adjusted**

**Table S6. Hazard ratios for primary outcome associated with current work schedule using Fine-Gray methods accounting for death as a competing risk in models**

**Table S7. Hazard ratios for primary outcome associated with current work schedule after excluding participants with follow-up time < 1 year or incident dementia <1 year from baseline**

**Table S8. Hazard ratios for primary outcome associated with current work schedule in the imputed dataset**

**Table S9. Hazard ratios for primary outcome associated with sleep duration**

**Table S1. Codes used in the UK Biobank study to identify dementia cases**

| **Dementia** | **ICD codes** | **Note** |
| --- | --- | --- |
| All-cause dementia | ICD-9 codes 290.2, 290.3, 290.4, 291.2, 294.1, 331.0, 331.1, 331.2, 331.5;  ICD-10 codes A81.0, F00, F01, F02, F03, F05.1, F10.6, G30, G31.0, G31.1, G31.8, I67.3. |  |
| AD | ICD-9 codes 331.0;  ICD-10 codes F00, G30. |  |
| VD | ICD-9 codes 290.4;  ICD-10 codes F01, I67.3. |  |
| Other types of dementia | ICD-9 codes 290.2, 290.3, 291.2, 294.1, 331.1, 331.2, 331.5;  ICD-10 codes A81.0, F02, F03, F05.1, F10.6, G31.0, G31.1, G31.8. | not AD or VD |

*Abbreviations: AD* Alzheimer’s disease, *ICD* International Classification of Diseases, *VD* Vascular dementia.

**Table S2. SNPs information for constructing polygenetic risk score**

| **Variants** | **Chr** | **Position** | **Closest gene** | **Major alleles** | **Minor alleles** | **MAF** | **OR** | **95% CI** | ***P* value** | **Included in PRS** |
| --- | --- | --- | --- | --- | --- | --- | --- | --- | --- | --- |
| rs4844610 | 1 | 207802552 | CR1 | C | A | 0.187 | 1.16 | 1.12-1.20 | 8.2*10-16 | 1 |
| rs6733839 | 2 | 127892810 | BIN1 | C | T | 0.407 | 1.18 | 1.15-1.22 | 4.0*10-28 | 1 |
| rs10933431 | 2 | 233981912 | INPP5D | C | G | 0.223 | 0.9 | 0.87-0.94 | 2.6*10-7 | 0 |
| rs190982 | 5 | 88223420 | MEF2C | A | G | 0.39 | 0.95 | 0.92-0.97 | 2.8*10-4 | 1 |
| rs9271058 | 6 | 32575406 | HLA-DRB1 | T | A | 0.27 | 1.1 | 1.06-1.14 | 5.1*10-8 | 0 |
| rs75932628 | 6 | 41129252 | TREM2 | C | T | 0.008 | 2.01 | 1.65-2.44 | 2.9*10-12 | 0 |
| rs9473117 | 6 | 47431284 | CD2AP | A | C | 0.28 | 1.09 | 1.05-1.12 | 2.3*10-7 | 1 |
| rs12539172 | 7 | 100091795 | NYAP1 | C | T | 0.303 | 0.93 | 0.91-0.96 | 2.1*10-5 | 1 |
| rs10808026 | 7 | 143099133 | EPHA1 | C | A | 0.199 | 0.9 | 0.87-0.94 | 3.1*10-8 | 1 |
| rs4723711 | 7 | 37844263 | NME8 | A | T | 0.356 | 0.95 | 0.92-0.98 | 2.7*10-4 | 0 |
| rs73223431 | 8 | 27219987 | PTK2B | C | T | 0.367 | 1.1 | 1.07-1.13 | 8.3*10-10 | 1 |
| rs9331896 | 8 | 27467686 | CLU | T | C | 0.387 | 0.88 | 0.85-0.91 | 3.6*10-16 | 1 |
| rs7920721 | 10 | 11720308 | ECHDC3 | A | G | 0.389 | 1.08 | 1.05-1.11 | 1.9*10-7 | 1 |
| rs3740688 | 11 | 47380340 | SPI1 | T | G | 0.448 | 0.91 | 0.89-0.94 | 9.7*10-11 | 0 |
| rs7933202 | 11 | 59936926 | MS4A2 | A | C | 0.391 | 0.89 | 0.86-0.92 | 2.2*10-15 | 1 |
| rs3851179 | 11 | 85868640 | PICALM | C | T | 0.356 | 0.89 | 0.86-0.91 | 5.8*10-16 | 1 |
| rs11218343 | 11 | 121435587 | SORL1 | T | C | 0.04 | 0.81 | 0.76-0.88 | 2.7*10-8 | 0 |
| rs17125924 | 14 | 53391680 | FERMT2 | A | G | 0.093 | 1.13 | 1.08-1.19 | 6.6*10-7 | 1 |
| rs12881735 | 14 | 92932828 | SLC24A4 | T | C | 0.221 | 0.92 | 0.88-0.95 | 4.9*10-7 | 0 |
| rs138190086 | 17 | 61538148 | ACE | G | A | 0.02 | 1.29 | 1.15-1.44 | 7.5*10-6 | 1 |
| rs3752246 | 19 | 1056492 | ABCA7 | C | G | 0.182 | 1.13 | 1.09-1.18 | 6.6*10-10 | 0 |
| rs429358 | 19 | 45411941 | APOE | T | C | 0.216 | 3.32 | 3.20-3.45 | 1.2*10-881 | 1 |
| rs6024870 | 20 | 54997568 | CASS4 | G | A | 0.088 | 0.88 | 0.84-0.93 | 1.1*10-6 | 0 |

*Abbreviation: SNPs* Single-nucleotide polymorphisms, *Chr* Chromosome, *MAF* Minor allele frequency, *OR* Odds ratio, *CI* Confidence intervals.

**Table S3. Hazard ratios for primary outcome associated with genetic predisposition to dementia by PRS category (n=170,722)**

|  | Genetic predisposition to dementia by PRS category | | | | | | |
| --- | --- | --- | --- | --- | --- | --- | --- |
|  | Low | Intermediate | | High | | *P* for trend | |
| Total cases | 221 | 189 | | 306 | | - | |
| Total sample size | 84855 | 43449 | | 42418 | | - | |
| Incidence Rate per 100,000 Person-Years | 21.28598 | 35.59999 | | 58.94839 | | *-* | |
| Person-Years | 1038242.0 | 530899.1 | | 519098.1 | | *-* | |
|  | Ref | HR (95% CI) | *P* | HR (95% CI) | *P* | HR (95% CI) | *P* |
| Model 1 | - | 1.61 [1.32, 1.95] | <0.001 | 2.84 [2.39, 3.38] | <0.001 | 1.69 [1.55, 1.84] | <0.001 |
| Model 2 | - | 1.59 [1.31, 1.94] | <0.001 | 2.84 [2.39, 3.37] | <0.001 | 1.69 [1.55, 1.84] | <0.001 |

*Abbreviation: PRS* Polygenetic risk score, *HR* Hazard ratios, *CI* Confidence interval. Model 1 was adjusted for age at baseline and sex. Model 2 was adjusted for terms in model 1, ethnicity, education, and socioeconomic status.

**Table S4. Incidence of primary outcome and secondary outcomes (n=170,722)**

|  |  | **Overall** | **Non-shift workers** | **Shift but non-night shift workers** | **Some night shift workers** | **Usual/permanent night shift workers** |
| --- | --- | --- | --- | --- | --- | --- |
|  | Sample size | 170722 | 143272 | 13729 | 7915 | 5806 |
| All cause dementia | Cases | 716 | 582 | 71 | 32 | 31 |
|  | Incidence Rate per 100,000 Person-Years | 34.28 | 33.19 | 42.40 | 33.11 | 43.67 |
|  | Person-Years | 2088239 | 1753205 | 167423 | 96623 | 70986 |
| AD | Cases | 271 | 223 | 25 | 12 | 11 |
|  | Incidence Rate per 100,000 Person-Years | 12.97 | 12.71 | 14.92 | 12.41 | 15.48 |
|  | Person-Years | 2089280 | 1754038 | 167545 | 96667 | 71028 |
| VD | Cases | 127 | 101 | 10 | 10 | 6 |
|  | Incidence Rate per 100,000 Person-Years | 6.07 | 5.75 | 5.96 | 10.34 | 8.44 |
|  | Person-Years | 2089609 | 1754274 | 167597 | 96689 | 71048 |
| Other types of dementia | Cases | 517 | 418 | 53 | 23 | 23 |
|  | Incidence Rate per 100,000 Person-Years | 24.75 | 23.83 | 31.64 | 23.79 | 32.38 |
|  | Person-Years | 2088804 | 1753631 | 167493 | 96651 | 71026 |

*Abbreviations: AD* Alzheimer’s disease, *VD* Vascular dementia.

**Table S5. Hazard ratios for primary outcome associated with current work schedule of models further adjusted (n=170,722)**

| **Sequency** | **Groups** | **Sample size** | **Cases** | **Person-Years** | **Incidence Rate per 100,000 Person-Years** | **Model 3** | **Model 4** | **Model 5** | **Model 6** | **Model 7** |
| --- | --- | --- | --- | --- | --- | --- | --- | --- | --- | --- |
| First | Non-shift workers | 143272 | 582 | 1753205 | 33.19 | Ref | Ref | Ref | Ref | Ref |
|  | Shift workers | 27450 | 134 | 335034 | 39.99 | 1.28 [1.06, 1.55] | 1.27 [1.05, 1.54] | 1.27 [1.05, 1.54] | 1.25 [1.03, 1.51] | 1.24 [1.02, 1.50] |
|  | *P* value | - | - | - | - | 0.01 | 0.015 | 0.015 | 0.025 | 0.032 |
| Second | Shift but non-night shift workers | 13729 | 71 | 167423 | 42.40 | Ref | Ref | Ref | Ref | Ref |
|  | Night shift workers | 13721 | 63 | 167610 | 37.58 | 1.04 [0.74, 1.47] | 1.05 [0.74, 1.48] | 1.06 [0.75, 1.50] | 1.04 [0.73, 1.48] | 1.03 [0.73, 1.47] |
|  | *P* value | - | - | - | - | 0.81 | 0.796 | 0.736 | 0.824 | 0.854 |
| Third | Some night shift workers | 7915 | 32 | 96623 | 33.11 | Ref | Ref | Ref | Ref | Ref |
|  | Usual/permanent night shift workers | 5806 | 31 | 70986 | 43.67 | 1.18 [0.72, 1.96] | 1.17 [0.70, 1.93] | 1.17 [0.70, 1.93] | 1.12 [0.68, 1.86] | 1.09 [0.65, 1.83] |
|  | *P* value | - | - | - | - | 0.51 | 0.551 | 0.552 | 0.651 | 0.736 |

Data are hazard ratios (95% confidence interval). Model 3 was adjusted for terms in model 2, diabetes mellitus, hypertension, stroke, coronary heart disease, cholesterol-lowering medication, antihypertensives, aspirin, body mass index, systolic blood pressure, total cholesterol, triglycerides, high-density lipoprotein, low-density lipoprotein, HbA1c, smoking status, alcohol consumption, healthy diet, and regular physical activity. Model 4 was adjusted for terms in model 3, genetic predisposition to dementia by polygenetic risk score category. Model 5 was adjusted for terms in model 4, years of work. Model 6 was adjusted for terms in model 5, sleep duration. Model 7 was adjusted for terms in model 6, chronotype preference.

**Table S6. Hazard ratios for primary outcome associated with current work schedule using Fine-Gray methods accounting for death as a competing risk in models (n=170,722)**

| **Sequency** | **Groups** | **Sample size** | **Cases** | **Death** | **Person-Years** | **Incidence Rate per 100,000 Person-Years** | **Model 1** | **Model 2** |
| --- | --- | --- | --- | --- | --- | --- | --- | --- |
| First | Non-shift workers | 143272 | 582 | 5221 | 1753205 | 33.19 | Ref | Ref |
|  | Shift workers | 27450 | 134 | 1122 | 335034 | 39.99 | 1.40 [1.16, 1.69] | 1.30 [1.07, 1.57] |
|  | *P* value | - | - |  | - | - | 0.00048 | 0.0066 |
| Second | Shift but non-night shift workers | 13729 | 71 | 603 | 167423 | 42.40 | Ref | Ref |
|  | Night shift workers | 13721 | 63 | 519 | 167610 | 37.58 | 1.09 [0.77, 1.54] | 1.04 [0.74, 1.47] |
|  | *P* value | - | - |  | - | - | 0.62 | 0.800 |
| Third | Some night shift workers | 7915 | 32 | 292 | 96623 | 33.11 | Ref | Ref |
|  | Usual/permanent night shift workers | 5806 | 31 | 227 | 70986 | 43.67 | 1.34 [0.82, 2.19] | 1.26 [0.76, 2.09] |
|  | *P* value | - | - |  | - | - | 0.240 | 0.370 |

Data are hazard ratios (95% confidence interval). Model 1 was adjusted for age at baseline and sex. Model 2 was adjusted for terms in model 1, ethnicity, education, and socioeconomic status.

**Table S7. Hazard ratios for primary outcome associated with current work schedule after excluding participants with follow-up time** **< 1 year or incident dementia <1 year from baseline (n=170,553)**

| **Sequency** | **Groups** | **Sample size** | **Cases** | **Person-Years** | **Incidence Rate per 100,000 Person-Years** | **Model 1** | **Model 2** |
| --- | --- | --- | --- | --- | --- | --- | --- |
| First | Non-shift workers | 143131 | 580 | 1753122.6 | 33.08 | Ref | Ref |
|  | Shift workers | 27422 | 134 | 335017 | 39.99 | 1.42 [1.17, 1.71] | 1.31 [1.08, 1.59] |
|  | *P* value | - | - | - | - | <0.001 | 0.005 |
| Second | Shift but non-night shift workers | 13714 | 71 | 167415 | 42.40 | Ref | Ref |
|  | Night shift workers | 13708 | 63 | 167602 | 37.58 | 1.09 [0.77, 1.53] | 1.04 [0.73, 1.47] |
|  | *P* value | - | - | - | - | 0.637 | 0.835 |
| Third | Some night shift workers | 7907 | 32 | 96619 | 33.11 | Ref | Ref |
|  | Usual/permanent night shift workers | 5801 | 31 | 70983 | 43.67 | 1.32 [0.81, 2.17] | 1.25 [0.76, 2.07] |
|  | *P* value | - | - | - | - | 0.266 | 0.374 |

Data are hazard ratios (95% confidence interval). Model 1 was adjusted for age at baseline and sex. Model 2 was adjusted for terms in model 1, ethnicity, education, and socioeconomic status.

**Table S8. Hazard ratios for primary outcome associated with current work schedule in the imputed dataset (n=278270)**

| **Sequency** | **Groups** | **Sample size** | **Cases** | **Person-Years** | **Incidence Rate per 100,000 Person-Years** | **Model 1** | **Model 2** |
| --- | --- | --- | --- | --- | --- | --- | --- |
| First | Non-shift workers | 230237 | 1035 | 2815207 | 36.76 | Ref | Ref |
|  | Shift workers | 48033 | 241 | 585254 | 41.17 | 1.34 [1.16, 1.54] | 1.24 [1.08, 1.43] |
|  | *P* value | - | - | - | - | <0.001 | 0.002 |
| Second | Shift but non-night shift workers | 23510 | 132 | 286421 | 46.08 | Ref | Ref |
|  | Night shift workers | 24523 | 109 | 298832 | 36.47 | 0.97 [0.75, 1.26] | 0.95 [0.73, 1.24] |
|  | *P* value | - | - | - | - | 0.867 | 0.739 |
| Third | Some night shift workers | 13810 | 56 | 168091 | 33.31 | Ref | Ref |
|  | Usual/permanent night shift workers | 10713 | 53 | 130741 | 40.53 | 1.22 [0.83, 1.78] | 1.16 [0.79, 1.71] |
|  | *P* value | - | - | - | - | 0.301 | 0.424 |

Data are hazard ratios (95% confidence interval). Model 1 was adjusted for age at baseline and sex. Model 2 was adjusted for terms in model 1, ethnicity, education, and socioeconomic status.

**Table S9. Hazard ratios for primary outcome associated with sleep duration (n=170,722)**

|  | Sleep duration | | | | |
| --- | --- | --- | --- | --- | --- |
|  | 7-8 hours | ≤6 hours | | ≥9 hours | |
| Total cases | 445 | 222 | | 49 | |
| Total sample size | 120237 | 42979 | | 7506 | |
| Incidence Rate per 100,000 Person-Years | 30.20 | 42.37 | | 53.69 | |
| Person-Years | 1473108 | 523873 | | 91257 | |
|  | Ref | HR (95% CI) | *P* | HR (95% CI) | *P* |
| Model 1 | - | 1.36 [1.16, 1.60] | <0.001 | 1.54 [1.15, 2.07] | 0.004 |
| Model 2 | - | 1.32 [1.12, 1.55] | 0.001 | 1.50 [1.12, 2.02] | 0.007 |

*Abbreviation: HR* Hazard ratios, *CI* Confidence interval.

Model 1 was adjusted for age at baseline and sex. Model 2 was adjusted for terms in model 1, ethnicity, education, and socioeconomic status.
